# The Interactive Association of General Obesity and Central Obesity with Prevalent Hypertension in Rural Lanzhou, China

**DOI:** 10.1371/journal.pone.0164409

**Published:** 2016-10-12

**Authors:** Wenlong Gao, Xiaowei Qiao, Yuhong Wang, Liping Wan, Zengwu Wang, Xin Wang, Zhaoxin Di, Xiaoyu Liu

**Affiliations:** 1 Institute of Epidemiology and Health Statistics, School of Public Health, Lanzhou University, Lanzhou, Gansu, P. R. China; 2 Lanzhou Center for Disease Prevention and Control, Lanzhou, Gansu, P. R. China; 3 Division of Prevention & Community Health, National Center for Cardiovascular Disease, Fuwai Hospital, Peking Union Medical College & Chinese Academy of Medical Sciences, Beijing, P. R. China; 4 Yuzhong County Center for Disease Prevention and Control, Lanzhou, Gansu, P. R. China; JAPAN

## Abstract

**Objective:**

To evaluate the interactive association between obesity with different anthropometry indices and prevalence of hypertension in rural Lanzhou.

**Methods:**

A cross-sectional survey was conducted in rural Lanzhou from April to July in 2013. The available information of 1275 rural residents aged more than 35 years was collected with a unified questionnaire and their blood pressure and anthropometry indices were measured in the field. The male-to-female ratio was 1:1.1. A generalized estimate equation (GEE) linear model was used to determine the association between obesity with different indexes and hypertension.

**Results:**

There was a moderate prevalence of general obesity (~11%) and very high prevalence of central obesity (53.2~67%) among the adults of rural Lanzhou. The prevalence of hypertension approximated 28%. GEE linear models showed that obesity with any one of anthropometry indices was associated significantly with the increased prevalence of hypertension among both males and females. In females, general obesity increased the prevalence of hypertension by 37% (0.37, 95%CI: 0.27,0.47) but in males by 23% (0.23, 95%CI: 0.12,0.35). The hypertensive effect of all central obesity was much lower than that of general obesity but approximately comparable to that of overweight. In addition, the interactions of the classified body mass index (BMI) and central obesity showed that when general obesity or overweight coexisted with any one of central obesity, the prevalence of hypertension was increased significantly, and this effect was a little higher than the corresponding main effect of general obesity or overweight in females but was much higher in males. In addition, general obesity or overweight which did not coexist with central obesity was not significantly associated with the increased prevalence of hypertension, nor were the other situations of central obesity in the normal weight or underweight except for the situation of central obesity with waist-to-hip ratio in the males of normal weight or underweight.

**Conclusion:**

In rural Lanzhou, higher BMI had larger associations with the increased prevalence of hypertension than central obesity indices. Only when general obesity or overweight coexisted with central obesity, the prevalence of hypertension was significantly increased. So, central obesity indices should be used jointly with BMI in evaluating the risk of hypertension.

## Introduction

Hypertension has been affirmed as the biggest single risk factor contributing to global death rates [1]. However, one-quarter of the world’s adult population is hypertensive and this proportion would increase to 29% by 2025 [2,3]. In China, the prevalence of hypertension in urban or rural areas continued to be increasing. Though the prevalence in the urban areas had always remained higher than that in the rural areas before 2006, the relative relation in prevalence between the two areas had switched after this year [3,4]. Obesity had been well recognized as the wide health risks including hypertension [5–7]. All the countries all over the world are searching for solutions to how to reverse the rise of obesity and member states of WHO have also proposed a voluntary target to stop the rise in obesity by 2025 [8]. How to win the battle is still a big challenge.

The certain anthropometric indices, such as body mass index (BMI), waist circumference (WC), waist-to-hip ratio (WHR), and waist-to-stature ratio (WSR) had been recommended to measure the body fat. Among these indices, BMI was the most widely used but unfortunately it was criticized for measuring body fat distribution inefficiently, especially abdominal fat mass. Some studies had shown that abdominal fat accumulation had a more critical correlation with the health risks than excess weight [9,10]. But, the capability of these obesity indices in predicting the hypertension risk still was debated [11–15]. These conflicting results showed it is necessary not only to continue exploring the effect of these obesity indices with hypertension among the population in different contexts but also to reveal the complex relationship between different types of obesity in prevalent hypertension.

In China, the cut-off values of the obesity classified with BMI and WC, which were different from the WHO standards, had been recommended by China Obesity Task Force, [16] and the well-accepted standard of WHR and WSR was used frequently to measure abdominal fat [17,18]. Some studies also revealed the association between these obesity indices and blood pressure level and attempted to determine which index was the optimal in predicting cardiovascular risk, but none of them measured the combined effect of general obesity and central obesity with their widely-used cut-off values on prevalence of hypertension. The current study was aimed to obtain the insights of optimizing the combined use of obesity indices for the prediction of hypertension risk in rural Lanzhou.

## Material and Methods

### Subjects and Settings

A cross-sectional survey was conducted in Yuzhong county of Lanzhou from April to July in 2013. Yuzhong County (one of three agricultural counties in Lanzhou) was pre-sampled randomly from all agricultural counties all over the country by Fuwai cardiovascular hospital. Other sample units such as the townships, the villages and households were obtained with the multi-stage systematic random sampling method. The sample size was estimated as a unit of county by means of a formula of the complete random sampling: n=uα22π(1−π)δ2, here,uα2=1.96, *π* is population rate and *δ* is a permissive error of sampling. A recent national report on nutrition and health survey showed an 18.8% prevalence of hypertension in the residents more than 18 years of age [19]. If population rate (*π*) was 18.8%, a permissive error(*δ*) was set at 0.125*π* and a non-response rate was 10%, total sample size was 1168 residents.So, no less than 1200 participants recruited all over the county could meet the need of the study. To control for the confounding impact of the constitution of age and sex, the participants were balanced in age and sex. The specific sampling procedure was that two townships were selected randomly out of the county, then two villages out of each sampled township; and then, no less than 300 residents aged more than 35 years were selected randomly out of each selected village. The selected residents were interviewed face-to-face with a pre-code self-designed questionnaire by trained professional interviewers from Lanzhou Center for Disease Control and Prevention and/or Yuzhong County Center for Disease Control and Prevention after he/she had signed the informed consent form. The questionnaire included the related information on the family socio-demographic characteristics, family socio-economic status, personal life style and behavior, diet, and the personal history of cardiovascular and metabolic diseases and the family history of hypertension. After the questionnaire was finished, their anthropometry indices, such as body height, body weight, WC, hip circumference (HC) and peripheral blood pressure, were measured by a trained doctor from the local township health center in the field. The right upper arm blood pressures of each participant were measured continuously 3 times with an interval of at least 30 seconds each time by mercury column type sphygmomanometer while he/she was sitting on a chair. The average of the 3-time measures was regarded as the actual blood pressure value. Body height and weight were also measured 2 times and their average values were recorded. WC was the circumference measure of 1 cm above the umbilicus level when the participant was breathing quietly. HC was recorded as the maximal circumference over the buttocks. All investigators and the personnel for quality control and data collection or entry were trained uniformly before the survey.

### Ethics Statement

We obtained the written informed consent forms from all the participants involved in the study after telling them about the process, purpose and confidentiality of the research. The study was reviewed and approved by the Ethics committee of Fuwai cardiovascular hospital affiliated with the Chinese Academy of Medical Sciences.

### Diagnosis of Hypertension

Hypertension was diagnosed by the hypertension criteria of WHO/ISH in 1999 [20]: systolic blood pressure was not lower than 140 mmHg and/or diastolic blood pressure were not lower than 90 mmHg. In addition, the participants with a history of hypertension (diagnosed in a county-or-above-level hospital) or taking antihypertensive drugs within 2 weeks regardless of whether their measured blood pressures were normal or not was identified as hypertension.

### Obesity Indexes and Diagnosis

The measured body height and weight were used to calculate the body mass index (BMI): BMI = weight/height^2^ (kg/m^2^). General obesity was identified with BMI more than 28 kg /m^2^, overweight with BMI of 24 -28kg /m^2^ and norm weight or underweight with BMI of ≤24 kg /m^2^ [16]. Central obesity was identified with WC (male: WC>85 cm or female: WC>80cm), [16] WHR (male: WHR≥0.9 or female: WHR≥0.85) [17] and WSR (WSR>0.5). [18] WHR = WC(cm)/HC(cm) and WSR = WC(cm)/height(cm). Central obesity with WC, WHR and WSR were called CO_WC_, CO_WHR_ and CO_WSR_ respectively in the current study.

### Study Variables

In the current study, hypertension and obesity with different anthropometric indices were the important study variables of interest. Taking into consideration that daily total oil or salt intake of the respondent may have a possible influence on hypertension, daily total oil or salt intake were evaluated by means of the following formula: daily intake (g/d) of plant (or animal) oil of the respondent = reported family monthly intake (an unit with 500g) of plant (or animal) oil×500g /(<family size-0.5×the number of children less than 6 years of age>×30d) and then daily total oil intake of the respondent (g/d) = daily plant oil intake (g/d) +daily animal oil intake (g/d); daily salt intake (g/d) = family monthly salt intake (an unit with 50g) ×50g /(<family size-0.5×the number of children less than 6 years of age>×30d). In addition, marital status, education, occupation, the average capita annual income of the family, smoking, drinking, drinking tea, the level of work intensity, having the diabetes and female menopausal status also were considered as the possible confounding factors in the evaluation of the relation between obesity and hypertension. As blood pressure is generally increasing over the course of life [21], the age of the participants was considered as an important control factor in the study.

### Data Analysis

Epidata 3.1 software (http://www.epidata.dk/) was used for dual data entry and SAS 9.2 software (Institute Inc. Cary, NC, USA) was adopted for statistical analysis. The measurement data was described using mean and standard deviation and the comparison between two means was made using Student’ *t*-test; the count data was described using rate or constitution ratio and chi-square test was used to compare the rate or constitution ratio between multiple groups. The trend of the prevalence of hypertension with age was tested using Z-test. In order to make the analysis robust, the participants with the missing or extreme values of anthropometry measures related to obesity indices and the missing values of sex were eliminated. Considering that the prevalence of hypertension may have a possible correlation in the same age of the participants, the generalized estimate equation (GEE) models were adopted to assess the association between obesity with different anthropometric indices and prevalence of hypertension while the possible correlation in the same age was controlled. For evaluating the interaction of classified BMI with any one of central obesity properly and taking the deficiency of the multiplication interaction in logistic regression model into consideration, GEE linear model with linear scale was adopted to evaluate the effects of classified BMI—general obesity, overweight and normal weight or underweight, central obesity with different indices and their additive interactions on hypertension [22]. In evaluating the main effects of classified BMI or central obesity with different indices, they were not included simultaneously in GEE linear models to avoid the collinearity [23,24]. To obtain the combined effect of classified BMI and central obesity, their combination were recoded as one variable with six values: general obesity+central obesity, general obesity+non-central obesity, overweight + central obesity, overweight + non-central obesity, normal weight or underweight+central obesity and normal weight or underweight+non-central obesity (reference), and it was entered together with all adjusted variables into GEE linear models. Finally, the main effects of classified BMI or central obesity with different indices and their additive interactions on hypertension were evaluated and their 95% confidence interval (CI) was computed in GEE linear models. All regression analyses were carried out separately for each sex. The statistical significance was set at 0.05.

## Results

### Population Characteristics and the Factors Related to Hypertension

Totally, 1325 participants were interviewed but in the study, the information of 1275 (S1 Data), including 610 males and 665 females, were available due to 19 missing values of body height, body weight, HC, WC and sex and 31 extreme values of the aforementioned four anthropometric indices. Table 1 depicts the socio-demographic characteristics and the factors related to hypertension by sex in the study population. Of all these participants, their average age was about 55 years, the majority was farmers, and a fairly large number had a low educational degree. The vast majority of them had been married but the males accounted for the higher proportion than the females. Less than one-fifth were engaged in the intense labor and this proportion among the males was a little higher than that among the females. Besides, the males were infatuated with smoking, drinking alcohol and tea much more frequently than the females. The family per capita annual income was generally less than 50000 RMB Yuan. For these participants, the average daily salt intake surpassed 8g. Approximately 3% of them suffered from diabetes and less than 20% of them had a family history of hypertension. Nearly two-thirds of the females had been in menopause.

**Table 1:** The basic characteristics and the related factors to hypertension.

Factors	Male		Female		χ^2^(*t*)	*P*-vale
	*n*	%	*n*	%		
Age(y)($\bar{X}$±SD)	55.29±11.40		54.82±10.71		0.76	0.45
35~	150	24.59	159	23.91		
45~	140	22.95	172	25.86		
55~	172	28.20	180	27.07		
65~	148	24.26	154	23.16		
Occupation(the farmer)	512	83.93	564	84.81	0.19	0.67
Educational level					155.78	<0.001
Primary school or below	35	5.76	202	30.42		
Junior high school	198	32.57	218	32.83		
Senior high school	248	40.79	193	29.07		
College or above	127	20.89	51	7.68		
Marital status(being married)	587	96.39	599	90.21	19.03	<0.001
Family per capita annual income(RMB_					4.69	0.32
<5000	171	28.03	173	26.02		
5000–10 thousand	151	24.75	178	26.77		
10–50 thousand	252	41.31	283	42.56		
>50 thousand	36	5.90	31	4.66		
Labor intensity(moderate or above)	113	18.52	86	12.93	7.55	<0.01
Smoking	394	64.59	1	0.15	617.86	<0.001
Drinking	184	30.26	8	1.21	208.49	<0.001
Drinking tea	332	54.43	75	11.28	272.56	<0.001
Daily oil intake (g/d) ($\bar{X}$±SD)	49.81±33.29		51.59±47.16		0.74	0.46
Salt intake(g/d($\bar{X}$±SD))	8.58±10.49		8.54±13.43		0.06	0.95
Having the diabetes	13	2.16	24	3.68	2.50	0.11
Family history of hypertension	118	19.34	111	16.69	1.52	0.22
Menopause	—	—	420	63.16		

SD: standard deviation

### Prevalence of Obesity with Different Anthropometric Indexes

**Fig 1:**
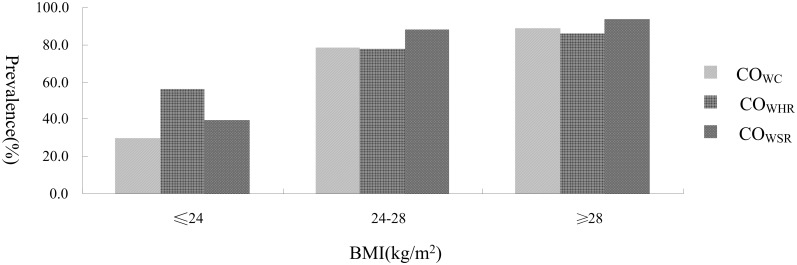
The BMI-specific prevalence of central obesity.

Table 2 shows the prevalence of obesity with different indices by sex. The overall prevalence of general obesity and overweight were 10.75% and 34.75% respectively. Of central obesity with different indices, the prevalence of CO_WHR_ was the highest (66.98%), to be followed by CO_WSR_ (62.12%) and CO_WC_ (53.18%). In addition, these prevalences in females were higher than those in males (general obesity:*P*<0.05;each of central obesity:*P*<0.001). Fig 1 shows the prevalence of central obesity with different indices in classified BMI. In those participants with general obesity or overweight, the prevalence of all central obesity surpassed 78% (overweight: 78.10–88.26%; general obesity: 86.13–93.43%). In addition, in those participants with BMI less than 24 kg/m^2^, the prevalence of CO_WHR_ (56.12%) was much higher than that of CO_WC_ (29.78%) or CO_WSR_ (39.28%).

**Table 2:** Sex-specific prevalence of obesity of different indexes among rural residents of Lanzhou.

	Male		Female		Overall		*χ*^2^	*P*-value
	*n*	%	*n*	%	*n*	%		
BMI(kg/m^2^)							8.14	<0.05
<24	357	58.52	338	50.83	695	54.51		
24–28	197	32.30	246	36.99	443	34.75		
≥28	56	9.18	81	12.18	137	10.75		
CO_WC_	267	43.77	411	61.80	678	53.18	41.56	<0.001
CO_WHR_	370	60.66	484	72.78	854	66.98	21.15	<0.001
CO_WSR_	320	52.46	472	70.98	792	62.12	46.37	<0.001

BMI: body mass index; CO_WC_: central obesity with waist circumference;CO_WHR_:central obesity with waist-hip ratio;CO_WSR_: central obesity with waist-stature ratio

### Prevalence of Hypertension

**Fig 2:**
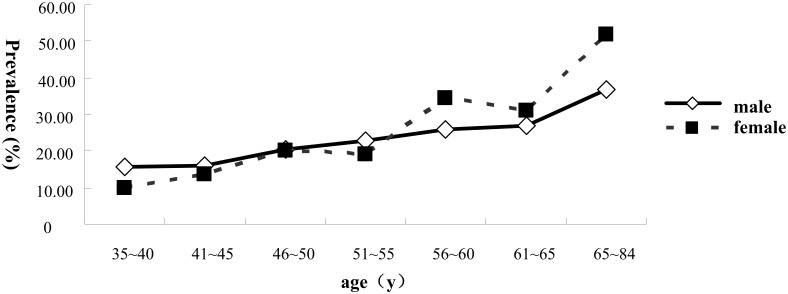
Age-specific prevalence of hypertension in both sexes.

The overall prevalence of hypertension was 27.76%. The prevalence of hypertension among the female (29.47%) was higher than that among the male (25.90%) but their difference was not significant statistically (*χ*^2^ = 2.08, *P* = 0.15). Fig 2 shows the age-specific prevalence of hypertension by sex. An obvious tendency that the prevalence of hypertension increased with age in both males and females (overall: Z = 2.05, *P* = 0.04) can be seen: before 50, the prevalence of hypertension in males was slightly higher than that of females; but after 50, prevalence of hypertension rose much more in females than in males.

### The Association between Obesity and the Prevalence of Hypertension

Table 3 shows the associations between obesity with different indices and the prevalence of hypertension in males and females by GEE linear regression models. While the possible correlation in the same age was controlled and all possible confounders were adjusted, the results of GEE models showed that obesity with any one of anthropometry indices was associated significantly with the increased prevalence of hypertension in both males and females. In females, general obesity increased the prevalence of hypertension by 37% (0.37, 95%CI: 0.27,0.47) but in males by 23% (0.23, 95%CI: 0.12,0.35). OW also had the increased effect on the prevalence of hypertension significantly (male: 0.15, 95%CI: 0.06,0.23; female: 0.08, 95%CI: 0.01,0.16), but its effect was far lower than that of general obesity and was higher in males than females. For 3 types of central obesity, in males the values of their effect on hypertension fell between 0.12 and 0.19 and in the female they lay between 0.12 and 0.15. Furthermore, the combined additive effects of the classified BMI and 3 types of central obesity were evaluated. When general obesity or overweight coexisted with any one of central obesity, the prevalence of hypertension was increased significantly, and in females this effect was a little higher than the corresponding main effect of general obesity or overweight but in males it was much higher. But, general obesity or overweight which did not coexist with central obesity was not significantly associated with the increased prevalence of hypertension, nor were the other situations of central obesity in the normal weight or underweight except for the situation of central obesity with waist-to-hip ratio in the males of normal weight or underweight.

**Table 3:** The association between classified BMI and central with different indexes on hypertension by GEE linear regression model.

	Male^a^			Female^b^		
	β	95%CI	*P*-value	β	95%CI	*P*-value
Main Effect						
BMI (kg/m^2^)						
≧28	0.23	0.12,0.35	< .001	0.37	0.27,0.47	< .001
24–28	0.15	0.06,0.23	< .001	0.08	0.01,0.16	< .05
<24	0			0		
CO_WC_	0.19	0.11,0.27	< .001	0.12	0.05,0.20	< .01
CO_WHR_	0.12	0.06,0.19	< .001	0.15	0.09,0.22	< .001
CO_WSR_	0.14	0.08,0.21	< .001	0.13	0.05,0.20	< .001
Additive Interaction						
BMI+CO_WC_						
≧28+CO_WC_	0.32	0.18,0.46	< .001	0.38	0.27,0.49	< .001
≧28+non-CO_WC_	-0.04	-0.24,0.16	.68	0.13	-0.09,0.35	.24
24–28+CO_WC_	0.20	0.11,0.30	< .001	0.10	0.01,0.19	< .05
24–28+non-CO_WC_	0.09	-0.07,0.24	.26	-0.04	-0.16,0.07	.45
<24+CO_WC_	0.12	0.002,0.24	< .05	-0.02	-0.10,0.07	.69
<24+non-CO_WC_	0			0		
BMI+ CO_WHR_						
≧28+CO_WHR_	0.34	0.19,0.48	< .001	0.43	0.32,0.55	< .001
≧28+non-CO_WHR_	0.01	-0.20,0.22	.95	0.28	-0.08,0.64	.12
24–28+CO_WHR_	0.20	0.10,0.29	< .001	0.16	0.08,0.24	< .001
24–28+non-CO_WHR_	0.08	-0.09,0.24	.37	0.03	-0.06,0.12	>.54
<24+CO_WHR_	0.05	-0.03,0.13	.26	0.08	-0.01,0.17	.07
<24+non-CO_WHR_	0			0		
BMI+CO_WSR_						
≧28+CO_WSR_	0.29	0.16,0.43	< .001	0.39	0.28,0.50	< .001
≧28+non-CO_WSR_	0.15	-0.06, 0.24	.18	0.40	-0.06,0.87	.09
24–28+CO_WSR_	0.18	0.09,0.26	< .001	0.11	0.03,0.19	<0.01
24–28+non-CO_WSR_	0.12	-0.08,0.32	.24	-0.01	-0.14,0.12	.88
<24+CO_WSR_	0.07	-0.04,0.17	.23	0.04	-0.07,0.14	.49
<24+non-CO_WSR_	0			0		

^*a*^: Twelve variables (marital status, education, occupation, the average capita annual income of the family, the level of work intensity, smoking, drinking, drinking tea, daily oil intake, daily salt intake, having the family history of hypertension and having the diabetes) were adjusted in GEE linear regression model;

^*b*^: ^*a*^-mentioned 12 variables and menopausal status were adjusted in GEE linear regression model.

BMI: body mass index; CO_WC_:central obesity with waist circumference;CO_WHR_:central obesity with waist-hip ratio;CO_WSR_:central obesity with waist-stature ratio;CI: confidence interval.

## Discussion

The current study found that general obesity was moderately prevalent but central obesity highly prevalent among the adults of rural Lanzhou. This finding was similar to the findings of the previous study in rural Hanzhong of western China [25]. Compared with the Gansu province-level prevalence in 2007 (Lanzhou is the capital of Gansu province in China), either general obesity or CO_WC_ had a large increase in prevalence [26]. However, the prevalence of hypertension in the current study had surpassed the level in 2010–2013 in rural China reported by a meta-analysis [3]. This implied that the parallel-growing trend observed over the last decade in the prevalence of obesity and hypertension across populations still remained in rural Lanzhou. The mutual increase of prevalent hypertension and obesity in the current study reflected the transition to urban lifestyles, dietary changes and lack of health education, for which a low healthcare coverage and inefficient health care system in rural China may take the responsibility [27]. Thus, the local government should pay more attention to this observed phenomenon that potential cardiovascular risk was still climbing in the rural community. The effective strategies should continue to be quested to attain their reverse through a series of special surveys on the cause of the prevalence and their increase. Meanwhile, the affordable and easily accessible primary health care system should be built by establishing community health centers in rural areas to realize the favorable ecology of medical care for rural residents [28].

GEE analysis for the main effect of classified BMI or central obesity with different indices showed that two types of obesity were significantly associated with the increased prevalence of hypertension among adult residents, regardless of their ages, in rural Lanzhou. This finding supported the stable relation between excess body fat and blood pressure. However, general obesity seemed to have a much larger prevalence of hypertension than any one of central obesity. This finding conformed to the result of the previous study of rural Hanzhong of western China [29]. It also implied that increased body mass may explain obesity-associated hypertension better than fat distribution in the study population. It is generally believed that the increased body mass would raise blood volume and cardiac output and then lead to the inadequate vasodilatation while the increased activity of the sympathetic nervous system, abnormal rennin-angiotensin-aldosterone relation and insulin resistance would arouse the defect in control of vascular resistance [21]. These adverse vascular responses may dominate the development of obesity-associated hypertension. However, central obesity seemed more likely to involve initially fat metabolic abnormality and then link to the insulin resistance [30]. Despite this, some previous studies also showed central-obesity-related indices like WC, WHR or WSR had a better predictive effect on hypertension [11–13]. Several researches also stressed that BMI best predicted hypertension [14,15]. However, in terms of the effect of obesity on cardiovascular risk, population-independency, race disparity, geographic difference and genetics susceptibility were common factors [31–33]. These complex influences may determine the diversity of the results in the studies with different contexts.

The current study found sex difference in the hypertensive effect of general obesity or overweight. General obesity in females seemed to foster the hypertension more frequently than that among the male, which may be due to a greater female responsiveness of blood pressure to general obesity [21]. Certainly, the female were naturally more susceptible to obesity compared with the male due to the reproductive transitions among women such as pregnancy and menopause, the physiological, emotional and psychosocial consequences suffered by women during the perimenopause [34,35]. Moreover these factors also induced the increase of their blood pressure to the certain extent [34]. The current study found the prevalence of obesity in females was indeed significantly higher than that in males and the prevalence of hypertension in females also seemed to be higher than that in males. A study showed that there was a consistent correlation between suboptimal health status and systolic blood pressure, total cholesterol, and high-density lipoprotein cholesterol in males and females, and that suboptimal health status is more prevalent in females than in males [36]. This may be another explicable reason of sex difference in hypertensive effect of obesity in the current study. In terms of the effect of overweight on hypertension, however, sex difference was just the opposite, which suggested the risk of hypertension in females was increased by a much larger increment with the rise of BMI than that in males. So, strict weight control appeared to be of great importance to females.

The combined effects of obesity with different anthropometry indices on hypertension, which had not been evaluated by any previous study in China, were evaluated by the current study. It was of crucial importance that highlighting the complete effect profile of obesity on hypertension would help to understand the hypertension risk caused by obesity accurately and determinate the priority of prevention and control strategies of obesity against hypertension. The results of GEE linear models for the interaction of classified BMI and central obesity showed that when general obesity or overweight coexisted with central obesity, the increased prevalence of hypertension was a little higher than the corresponding main effect of general obesity or overweight in females but was much higher in males. The previous study revealed that body fat distribution could not modulate the relation between obesity and blood pressure due to the strong inter-correlations of obesity indices, [21]and the current study did find that those participants with general obesity or overweight had the extremely high prevalence of central obesity (overweight: 78.10–88.26%; general obesity: 86.13–93.43%). But the hypertensive effect of general obesity seemed to be enhanced by combined central obesity in the current study and the increased effect in males was more obvious than that in females. The current study also found that central obesity among the normal or underweight participants, except for CO_WHR_ in males, did not seem to be associated with the risk of hypertension. On the other hand, central obesity was quite prevalent among those normal or underweight participants in the current study, which may be attributed to that greater predisposition of abdominal obesity and visceral fat made some people increase abdominal obesity with a lower BMI [37]. However, this central obesity may not increase substantially the risk of hypertension in most cases. In addition, general obesity+non-central-obesity or overweight+non-central obesity also was not associated with the increased prevalence of hypertension but general obesity+central obesity or overweight + central obesity was, which implied that the cooperation of relative weight gain and abdominal fat accumulation was more critical and important mechanism in the development of obesity-associated hypertension than the separate single effect of their own. This is mainly explained that most of obese individuals had the extremely high possibility of the coexisting of relative weight gain and abdominal fat accumulation due to many shared pathophysiological changes. So, comprehensive prevention strategies against hypertension should lay much emphasis on the priority of the intervention of the people with excess weight and central distribution of adiposity. In addition, all the results of the interaction of classified BMI and central obesity also seemed to agree with other researches that the combined use of BMI and central-obesity measures was better predictors of hypertension than BMI or central-obesity indices alone [38,39]. Therefore, BMI and central-obesity indices should be used jointly in assessing the risk of hypertension. In contrast to central-obesity indices, WHR may have a larger advantage in the combined use of BMI as a screening tool than WC or WSR because its proportion in the individuals with BMI less than 24 kg/m^2^ was the highest and CO_WHR_ in the normal or underweight males had a weak association with risk of hypertension. The current study is, of course, not without limitations. So, further research is needed to understand the mechanism underlying the observed phenomena.

Several limitations need to be acknowledged. First, the current study is a cross-sectional survey, which does not determine the causal relationship between obesity and hypertension. Second, all adjusted variables were collected on the basis of participants’ recall, so recall bias could not be avoided. Third, the substantial absolute errors in measuring waist and hip circumferences may exist [15]. So, the latent measurement errors may affect the evaluation of central obesity and then influence the hypertensive effect related to central obesity.

In conclusion, in rural Lanzhou general obesity was moderately prevalent but central obesity highly prevalent. Higher BMI had larger associations with the increased risk of hypertension than any one of central-obesity indices. General obesity or overweight coexisting with central obesity had the higher risk of hypertension, but general obesity or overweight without central obesity and central obesity in normal weight or underweight did not seem to. So, central-obesity indices should be used jointly with BMI in evaluating the risk of hypertension.

## Supporting Information

S1 File Supporting Data (ZIP)
